# Molecular and histological validation of modified *in ovulo* nucellus culture based high-competency direct somatic embryogenesis and amplitude true-to-the-type plantlet recovery in Kinnow mandarin

**DOI:** 10.3389/fpls.2023.1116151

**Published:** 2023-03-08

**Authors:** Theivanai Murugan, Om Prakash Awasthi, Sanjay Kumar Singh, Gautam Chawla, Amolkumar U. Solanke, Sunil Kumar, Girish Kumar Jha

**Affiliations:** ^1^ Division of Fruits and Horticultural Technology, ICAR-Indian Agricultural Research Institute, New Delhi, India; ^2^ Division of Nematology, ICAR-Indian Agricultural Research Institute, New Delhi, India; ^3^ Division of Molecular Biology and Biotechnology, ICAR-National Institute for Plant Biotechnology, New Delhi, India; ^4^ Discipline of Agricultural Statistics, Division of Agricultural Economics, ICAR-Indian Agricultural Research Institute, New Delhi, India

**Keywords:** DKW, fruit developmental stages, ISSR clonal fidelity, Kinnow, ovule developmental events, ovule histology, somatic embryogenesis

## Abstract

Kinnow (*Citrus nobilis* Lour. × *Citrus deliciosa* Ten.) needs to be genetically improved for traits such as seedlessness using biotechnological tools. Indirect somatic embryogenesis (ISE) protocols have been reported for citrus improvement. However, its use is restricted due to frequent occurrences of somaclonal variation and low recovery of plantlets. Direct somatic embryogenesis (DSE) using nucellus culture has played a significant role in apomictic fruit crops. However, its application in citrus is limited due to the injury caused to tissues during isolation. Optimization of the explant developmental stage, explant preparation method, and modification in the *in vitro* culture techniques can play a vital role in overcoming the limitation. The present investigation deals with a modified *in ovulo* nucellus culture technique after the concurrent exclusion of preexisting embryos. The ovule developmental events were examined in immature fruits at different stages of fruit growth (stages I–VII). The ovules of stage III fruits (>21–25 mm in diameter) were found appropriate for *in ovulo* nucellus culture. Optimized ovule size induced somatic embryos at the micropylar cut end on induction medium containing Driver and Kuniyuki Walnut (DKW) basal medium with kinetin (KIN) 5.0 mg L^-1^ and malt extract (ME) 1,000 mg L^-1^. Simultaneously, the same medium supported the maturation of somatic embryos. The matured embryos from the above medium gave robust germination with bipolar conversion on Murashige and Tucker (MT) medium + gibberellic acid (GA_3_) 2.0 mg L^-1^ + ά-naphthaleneacetic acid (NAA) 0.5 mg L^-1^ + spermidine 100 mg L^-1^ + coconut water (CW) 10% (v/v). The bipolar germinated seedlings established well upon preconditioning in a plant bio regulator (PBR)-free liquid medium under the light. Consequently, a cent percent survival of emblings was achieved on a potting medium containing cocopeat:vermiculite:perlite (2:1:1). Histological studies confirmed the single nucellus cell origin of somatic embryos by undergoing normal developmental events. Eight polymorphic Inter Simple Sequence Repeats (ISSR) markers confirmed the genetic stability of acclimatized emblings. Since the protocol can induce rapid single-cell origin of genetically stable *in vitro* regenerants in high frequency, it has potential for the induction of solid mutants, besides crop improvement, mass multiplication, gene editing, and virus elimination in Kinnow mandarin.

## Introduction

Citrus is rated as a high-value fruit crop known for its refreshing juice, vitamin C content, secondary metabolites, and high antioxidant properties. So immense are the nutritional values that it was recommended as an immunity booster during the pandemic (WHO, 2021). These desirable attributes signify the glory of this fruit in world commerce by an export quantum of 1.7 million tonnes of fruits per annum. Globally, citrus is grown in an area of 10.07 million hectares (ha) with a production of 143.75 million tonnes. Although sweet orange dominates more than 50% of global citrus production, mandarin and its groups dominate India’s citrus industry by accounting for more than 43% of production (FAO, 2021). Among mandarins, Kinnow, an interspecific hybrid introduced from California, USA, made a breakthrough in the citrus industry of India and has established itself as the most preferred fresh fruit among consumers. There is a great demand for Kinnow fruits for processing into juice. However, it could not get a boost due to its high seediness leading to delayed bitterness (Mallick et al., 2016; Kumar et al., 2021). The development of low-seeded scion varieties is a major target for citrus breeders worldwide, which, however, is difficult to achieve through conventional breeding.

Modern breeding approaches such as *in vitro* mutagenesis have opened new vistas for achieving the traits of interest over *ex vitro* mutagenesis (Penna et al., 2012). In this direction, solid mutant induction can be considered a powerful speed breeding tool in perennial fruit crops. The totipotency of single cells can be utilized to induce solid mutants through *in vitro* techniques. Since indirect somatic embryogenesis (ISE) and direct somatic embryogenesis (DSE) play an essential role in single-cell regeneration, these techniques have immense value in citrus species (Omar et al., 2016). Standard ISE protocols in citrus using female reproductive organs such as stigma, style, ovary, and ovule are available (Gmitter and Moore, 1986; Cardoso et al., 2012; Omar et al., 2016). Earlier workers have suggested ISE from undeveloped ovules as the most effective protocol for citrus improvement (Grosser et al., 2000; Dutt et al., 2010; Ge et al., 2015; Omar et al., 2016). Contrary to the recommendations given by earlier workers, few researchers have also reported the frequent occurrence of somaclonal variations from ISE. Culture-induced somaclonal variation is undesirable when efforts are underway to produce genetically identical regenerants (Hao and Deng, 2002; Grosser et al., 2007; Dutt and Grosser, 2010; Meziane et al., 2017). Similarly, the ISE system, although has been reported to induce high-frequency somatic embryogenesis, *ex vitro* characterization of obtained plantlets is difficult due to the low recovery of hardened plantlets (Grosser and Gmitter, 1990; Fleming et al., 2000). Recent studies emphasize the importance of DSE for overcoming the somaclonal variation as well as rapid mass multiplication and high plantlet recovery in different fruit crops such as banana, citrus, date palm, mango, and passion fruit (Kochba et al., 1972; Navarro and Juarez, 1977; Panis et al., 1993; Wu et al., 2007; Silva et al., 2021).

The DSE technique holds promise particularly in polyembryonic fruit crops. In some citrus species, the sporophytic mixed apomixis type of polyembryony has been reported and contains both zygotic and nucellar embryos within the same seed (Bewley et al., 2012). Polyembryony in citrus is referred to as nucellar embryony because of its nucellus origin (Kepiro and Roose, 2007). For achieving *in vitro* DSE, nucellus tissue can be utilized by taking advantage of predetermined embryogenic and primordial cells in nucellar embryonic varieties. It leads to embryo development from a single cell to fulfill the need of its usage in solid mutant induction (Bitters et al., 1972; Kochba et al., 1972; Esen and Soost, 1977; Navarro and Juarez, 1977; Kobayashi et al., 1979). From a mutagenesis perspective, removing the embryos before explant inoculation is a prerequisite to avoiding zygotic variation and chimera formation. Migration of embryos toward the micropylar end after pollination is the characteristic feature of nucellar embryony, and the duration of its complete migration varies among citrus species (Wakana and Uemoto, 1987). Therefore, histological identification of explant developmental stages with probable complete migration of embryos toward the micropylar end must be standardized to confirm the absence of embryos from explants before inoculation.

Mandarins are parents for many citrus species of polyembryonic origin. It is well-established that mandarin varieties are recalcitrant and give poor *in vitro* regeneration response (Gmitter and Moore, 1986; Tomaz et al., 2001; Ricci et al., 2002; Germana, 2003; Benelli et al., 2010). The wild and primitive nature may be the reason for a few of its varieties to respond poorly under *in vitro* culture conditions. Furthermore, the interspecific hybridity of Kinnow (*Citrus nobilis* × *Citrus deliciosa*) makes embryogenesis complex (author’s unpublished data). Although nucellus cultures can induce DSE in citrus, the injury caused during tissue excision gives a lower success (Bitters et al., 1972; Kochba et al., 1972; Navarro and Juarez, 1977). Since the technique has an immense advantage for obtaining high recovery of solid mutants, the present study is an attempt toward modification of protocol through *in ovulo* nucellus culture for getting genetically stable regenerants with high plantlet recovery from nucellus tissue in Kinnow mandarin. The study also includes histological examination and analysis of clonal fidelity employing the ISSR marker system for seeking histological and molecular evidence for the proposed protocol’s robustness.

## Materials and methods

### Plant material

The mother plants of Kinnow mandarin maintained at a spacing of 6 m × 6 m in the Experimental Orchard of Division of Fruits and Horticultural Technology, ICAR-IARI, New Delhi, India (latitude 28°38ʹ23ʺN, longitude 77°09ʹ27ʺE) were used as a source for fruit collection. The plants were maintained following the recommended cultural practices. During the first year of experimentation (2019), the fruit size and the corresponding developmental events in the ovule were examined for explant excision. The aim was to identify the ideal ontogenic stage of developing fruits to get an appropriate ovule explant for high-frequency embryogenesis from nucellus tissue. The experiment was repeated in the 2020 season for conformity, and standardization of the somatic embryogenesis protocol was taken up.

### Standardization of optimum fruit size for explant collection

Immature cross-pollinated fruits of Kinnow mandarin were collected between 30 and 150 days having fruit diameters between 5 and 45 mm. Uniform-sized fruits were collected at staggered intervals from different canopy directions and shifted to the laboratory in an ice box. Fruits from each collective interval were sorted for uniformity following measurement with Vernier calipers (Mitutoyo digital caliper, China). The fruits were then rinsed in running tap water and kept on a blotting paper to remove excess surface moisture. Surface sterilized fruits were cut into two halves, and well-developed bold ovules were collected carefully. The ovules were then dissected under a Zeiss SteREO Discovery V8 stereo zoom digital microscope (Goettingen, Germany) to observe and measure developmental events as outlined by Bitters et al. (1972). The same procedure was repeated until fruits attained 45-mm diameter and were grouped into seven stages based on ovule internal developmental events with corresponding fruit size. Finally, the optimum fruit size was determined based on the ovule length, embryo coverage, and presence of a liquid endosperm surrounded by a prominent nucellus. The experiment was repeated thrice with 25 random samples per replication.

### Explant preparation

Stage III fruits were surface sterilized with 0.5% Bavistin^®^ (carbendazim) and 0.5% Ridomil^®^ (mancozeb + metalaxyl) for explant preparation. One hour after fungicide treatment, the fruits were taken into the laminar air-flow chamber and rinsed twice with sterile double-distilled water, immersed in ethanol, and flame sterilized for a few seconds. Following these procedures, fruits were cut open with sterile forceps and scalpels. The ovules were then collected in a sterile Petri dish exercising all care to avoid damage incurred to the ovules. Based on stereomicroscopic observations, the micropylar end containing zygotic and nucellar embryos was removed using a sterile razor blade with a fine cut. The prepared ovule explants were immediately inoculated by placing the chalazal end on the induction cum maturation (ICM) medium to avoid desiccation.

### Somatic embryo induction and maturation

For induction and maturation of somatic embryos from Kinnow *in ovulo* nucellus explant, different basal media, i.e., Murashige and Tucker (MT) (Murashige, 1969), Driver and Kuniyuki Walnut (DKW) (Driver and Kuniyuki, 1984), and Gamborg B_5_ medium (B_5_) (Gamborg et al., 1968), supplemented with plant bio regulators (PBRs) (auxins and cytokinins) in combination with organic additives such as malt extract (ME) and coconut water (CW) were tried. The treatment combinations for ICM are EM_1_ (embryogenic medium): Control, EM_2_: ME 500 mg L^-1^, EM_3_: kinetin 5.0 mg L^-1^ + ME 500 mg L^-1^, EM_4_: kinetin 5.0 mg L^-1^ + ME 1,000 mg L^-1^, EM_5_: 2,4-dichlorophenoxyacetic acid (2,4-D) 1.0 mg L^-1^ + CW 10% (v/v), and EM_6_: 2,4-D 2.0 mg L^-1^ + CW 10% (v/v). Stereomicroscopic observations were made at monthly intervals to record percent somatic embryogenic response, days to embryogenesis, whereas the number of somatic embryos per explant was counted 4 months after initiation. Subculturing was carried out at an interval of 2 months on the same medium composition for proliferation and maturation. Cultures were maintained under dark with 25°C ± 2°C temperature and 70%–80% Relative humidity (RH). The experiment was replicated thrice with 25 samples in each replication.

### Somatic embryo germination and conversion

Cotyledonary somatic embryos (>4 mm in length) of SE_1_ (somatic embryos): MT × EM_4_, SE_2_: DKW × EM_4_, and SE_3_: B_5_ × EM_4_ were transferred to germination cum conversion (GCC) medium to compare the effect of ICM medium composition on germination and conversion. The details of GCC treatments include GM_1_ (germination medium): Basal MT, GM_2_: MT + ME 500 mg L^-1^, GM_3_: MT + GA_3_ 2.0 mg L^-1^ + ά-naphthaleneacetic acid (NAA) 0.5 mg L^-1^ + spermidine 100 mg L^-1^ + CW 10% (v/v), GM_4_: DKW + GA_3_ 2.0 mg L^-1^ + NAA 0.5 mg L^-1^ + spermidine 100 mg L^-1^ + CW 10% (v/v), GM_5_: DKW + kinetin 5.0 mg L^-1^ + ME 1,000 mg L^-1^, and GM_6_: B_5_ + 6-benzylaminopurine (BAP) 2.0 mg L^-1^ + NAA 0.2 mg L^-1^ + GA_3_ 0.5 mg L^-1^ + abscisic acid (ABA) 0.2 mg L^-1^ + activated charcoal (AC) 200 mg L^-1^ + CW 10% (v/v). The experiment was repeated thrice, and data were recorded on percent germination, days to germination, and percentage of bipolar seedling conversion. Cultures of each replication containing 25 samples were maintained under aseptic culture conditions at 25°C ± 2°C temperature and 70%–80% RH under dark until germination.

### Plantlet establishment and acclimatization

Plantlet establishment frequency was tested by transferring germinated bipolar seedlings of >4 cm long from highly responsive GCC media interactions (SE_1_ × GM_1_, SE_1_ × GM_3_, SE_1_ × GM_4_, SE_2_ × GM_1_, SE_2_ × GM_3_, SE_2_ × GM_4_, SE_3_ × GM_1_, SE_3_ × GM_3_, SE_3_ × GM_4_) to PBR-free solid and liquid MT media under light [photoperiod of 16/8 light/dark cycle (26.81 µmol m^-2^ s^-1^)]. To support plantlets on a liquid medium, a sterile Whatman^®^ filter paper bridge was used. For primary hardening, established 4–5-leaf stage emblings of GCC_9_ from both solid and liquid media were removed, washed thrice with sterile double-distilled water, followed by its immersion in 0.1% Bavistin solution for 5 min and then transferred to different potting media in 250-ml culture bottles [P_1_, cocopeat:vermiculite:perlite (2:1:1); P_2_, cocopeat:vermiculite (2:1); and P_3_, cocopeat:perlite (2:1)]. The data on percent survival and shoot and root length were recorded on the 60th day, while the emergence of new leaves was recorded at a periodic interval. During acclimatization, a half-strength liquid medium without sucrose was sprayed on the emblings. When shoot growth reached the bottle top, the culture bottle caps were loosened gradually to expose the emblings to an ambient environment. The observations were recorded from 10 samples per replication in the above experiments.

### Media preparation

After adding the required media components, 50 g L^-1^ sucrose and 7 g L^-1^ agar were added, pH was adjusted between 5.7 and 5.8 with 1N HCl and KOH, then autoclaved at 121°C (15 psi) for 20 min. Plant growth regulators and CW were added to autoclaved media at a lukewarm temperature, and ME was added before autoclaving. Potting media were sterilized for 1 h at 121°C (15 psi). The ready-to-use plant tissue culture-grade MT medium, DKW medium, and B_5_ were procured from Himedia^®^, Maharashtra, India. Similarly, inorganic salts, organic ingredients, sucrose, agar, and AC used for culture media were also purchased from Himedia^®^. Whereas growth regulators of cell culture tested grade were used from Sigma Chemicals Co., USA, and Central Drug House (P) Ltd., New Delhi, India.

### Histological analysis

For histological observation, 15 ovule samples (five ovules/replicate) at the time of explant collection and post *in vitro* inoculation were fixed overnight in FAA [5:5:90 formalin (37%–40%), glacial acetic acid, and ethyl alcohol (50% v/v)] (Yeung and Saxena, 2005). The fixed tissue samples were dehydrated in ethanol series varying from 40% to 100% and were then cleared in ethanol:xylene (3:1, 1:1, and 1:3) for 30 min each and left overnight. Dehydration with 100% ethanol and tissue clearing in the ratio of 1:3 were repeated thrice and twice, respectively. After impregnation, i.e., by a gradual increase of wax concentration in samples and its subsequent transfer in absolute molten wax (four times), samples were embedded in paraffin wax. Following embedding, 12-µm sections were prepared using a hand-operated rotatory microtome (MAC) and stained with safranin and fast green for 2 min between each ethanol series following the procedure suggested by Moreno-Sanz et al. (2020) with minor modifications. The sections were observed under Zeiss Axio Imager M2m and photographed using a 5-megapixel high-resolution camera (Axio Vision).

### Fidelity test

DNeasy^®^ Plant Mini Kit (Qiagen) was used for genomic DNA isolation from leaf samples of randomly selected five *in vitro* primary hardened Kinnow regenerants along with *in vivo* grown mother plant. Qualitative and quantitative analyses of isolated DNA were carried out using gel electrophoresis. As Pal et al. (2013) outlined, 10 ISSR primers were used for initial screening based on the optimized protocol by Murugan et al. (2022). The PCR reaction mixture of 25 µl consisting of 10× buffer (2.5 µl), 10 mM dNTPs (2.0 µl), 5 U/µl *Taq* DNA polymerase (0.3 µl), 1 U primer (1.0 µl), (30 ng µl^-1^) DNA of 1.5 µl, and deionized water (17.7 µl) was prepared. PCR reactions of 35 cycles with an initial denaturation at 95°C (5 min), annealing at 50°C (30 s), primer extension at 72°C (2 min), and final extension at 72°C (10 min) were carried out using a thermal cycler (Eppendorf Master Cycler, Hamburg, Germany). Electrophoresis was done in 2.0% agarose gel stained with ethidium bromide (EtBr) using 1× Tris-acetate-EDTA (TAE) buffer (pH 8.0) at 120 V for 3 h to resolve PCR amplification products. For visualization, scanning, and photography of gel, gel documentation (Bio-Rad Gel Doc XR^+^) was used. Reproducible bands were scored regardless of their intensities, and those with the same migration were considered monomorphic. The experiment was repeated thrice for reproducibility.

### Statistical analysis

The *in vitro* experiments were analyzed using one-way ANOVA with two factors to test the significance of mean differences in completely randomized design (CRD). Each experiment was replicated thrice, and a different number of samples was observed in each replication, as mentioned in the subsections above. Analysis was performed using RStudio, Version 4.2.0 (RStudio Inc., Boston, MA, USA). The percentage data were transmuted to arcsine before ANOVA. Duncan’s multiple range test (DMRT) was used to compare the treatment means at P < 0.05 significance level.

## Results

### Identification and classification of ovule developmental stages

**Figure 1:**
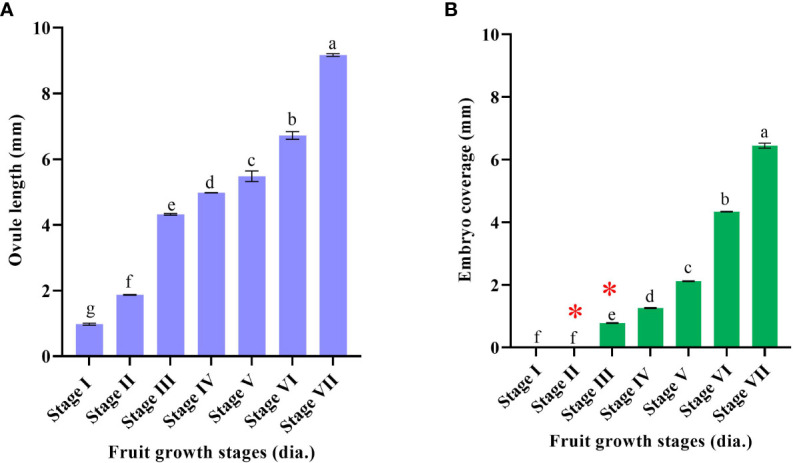
Fruit diameter and corresponding ovule developmental events in Kinnow mandarin. **(A)** Ovule length (mm), **(B)** embryo coverage: * represents the presence of a liquid endosperm. Stage I (5–14 mm), stage II (>14–21 mm), stage III (>21–25 mm), stage IV (>25–28 mm), stage V (>28–34 mm), stage VI (>34–38 mm), and stage VII (>38 mm) immature fruit diameter. Small letters denote the statistical difference; the same letters represent non-significant differences in the mean values.

**Figure 2:**
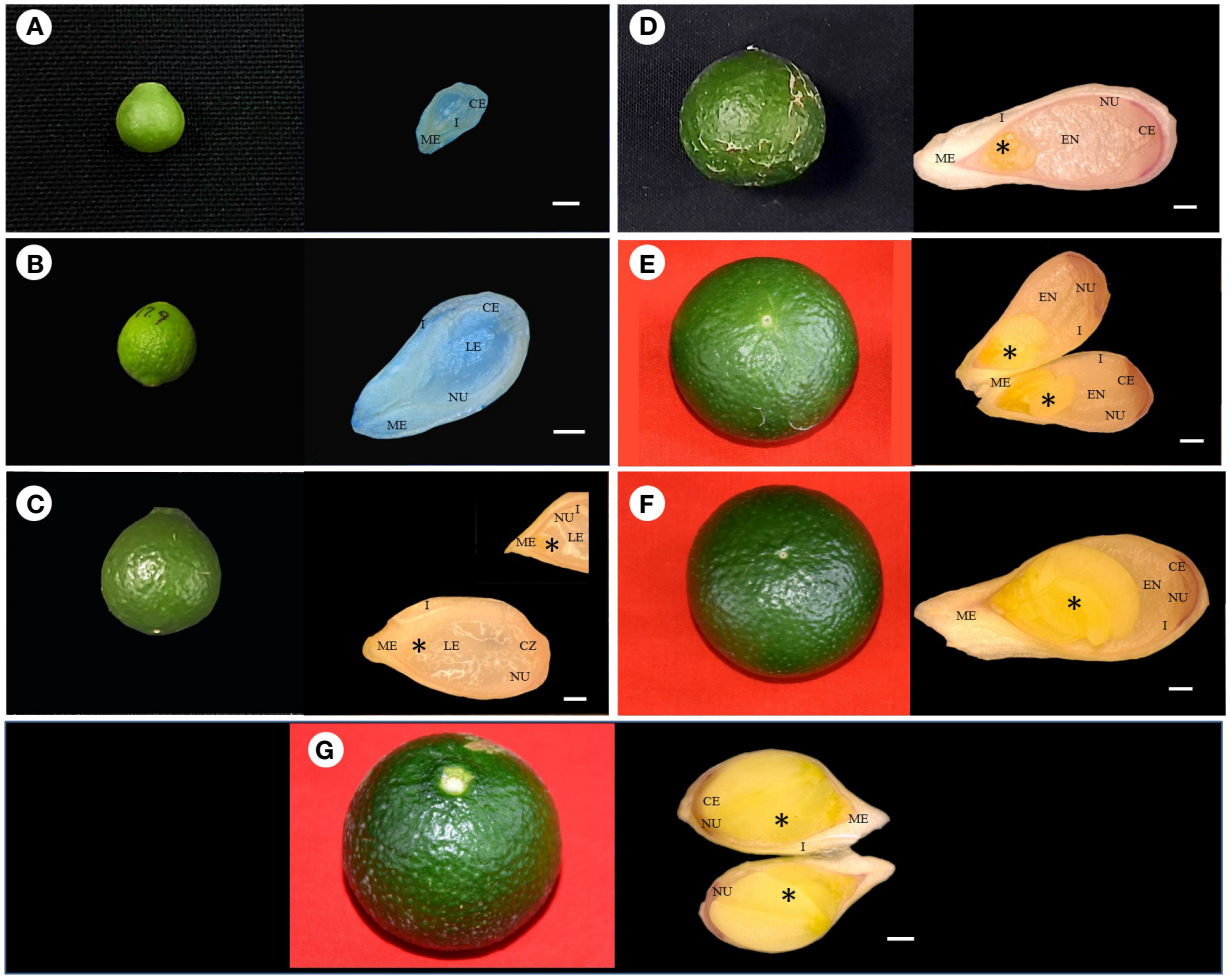
Fertilized ovule developmental stages of corresponding fruit size in Kinnow mandarin. **(A)** Ovule of 5-mm-diameter fruit, **(B)** ovule of 19-mm-diameter fruit, **(C)** ovule of 24-mm-diameter fruit, **(D)** ovule of 26.8-mm-diameter fruit, **(E)** ovule of 33-mm-diameter fruit, **(F)** ovule of 36-mm-diameter fruit, **(G)** ovule of 40-mm-diameter fruit. Scale bar represents 500 µm, *represents embryo. CE, chalazal end; EN, endosperm; I, integuments; LE, liquid endosperm; ME, micropylar end; NU, nucellus tissue.

Stereomicroscopic observations during the post-anthesis fruit developmental stages (5–45 mm in diameter) revealed interesting results. Based on the developmental events of ovule corresponding to fruit diameter, the following classifications were made, *viz.*, stage I (5–14 mm), stage II (>14–21 mm), stage III (>21–25 mm), stage IV (>25–28 mm), stage V (>28–34 mm), stage VI (>34–38 mm), and stage VII (>38 mm) (**Figures 1A, B** **)**. Fruits measuring 5–14 mm in diameter had an ovule size of <2 mm (stage I) (**Figures 1**, **2A** **)**. In the subsequent fruit development stages, >14–21-mm-diameter fruits [50–70 days after anthesis (DAA)] showed faster ovule growth and measured approximately 2–3 mm (stage II). At this stage, the ovule had an intact nucellus and the presence of a liquid endosperm. However, the embryos were not visible (**Figures 1**, **2B**). When the fruits attained a size of >21–25 mm in diameter (75–110 DAA), ovules grew to a size of more than 4 mm (stage III). Compared to the initial two stages, where dissection and handling were difficult, it was much easier to handle and dissect the stage III samples. The ovule color at stage III was pale white, containing a liquid endosperm, intact nucellus, and nucellus covering the entire embryo sac. Multiple globular to heart-shaped embryos could be seen crowded at the micropylar end of the embryo sac, while no visible embryo differentiation was witnessed in nucellus tissue (**Figures 1**, **2C**). The stage IV fruits measuring >25–28 mm in diameter (110–120 DAA) contained ovules (<5 mm) with a semisolid endosperm. The nucellus at this stage started depleting from the micropylar end, and a few embryos were converted to torpedo and cotyledonary stages. In contrast, at the chalazal end, the pink color appeared (**Figures 1**, **2D**). At stage V, when the fruits were >28–34 mm in diameter, the ovules grew to a length of 5–5.5 mm and the embryo occupied nearly half of the embryo sac, while the nucellus was visible at the chalazal half (120–130 DAA) (**Figures 1**, **2E**). More than 35-mm fruits of stage VI had embryo growth extended to ¾ the size of ovules (6–7 mm) (**Figures 1**, **2F**), and the nucellus was visible at the chalazal end (135 DAA). Stage VII fruits beyond 39 mm (140 DAA) showed ovules of >8 mm, where the embryo covered the maximum portion of the embryo sac by depleting the endosperm (**Figures 1**, **2G**).

The complete depletion of the nucellus occurred nearly 150 DAA. Hence, this particular experiment was conducted for up to 150 days. Initial immature fruit developmental stages revealed three nonsequential orders in fruit growth and ovule growth. Firstly, ovule size was nearly similar, but fruit growth was faster, followed by *vice versa* in the second order, and then in the third round, both ovule and fruit had faster growth. It was evident from the above result that fruits of 5–21 mm in diameter have ovules of <3 mm (up to 70 DAA) in size, whereas 21–25-mm fruits have ovule lengths of >4 mm (after another 35 days) followed by visible changes observed in the ovule and fruit growth at a faster rate in upcoming growth stages (40 days). However, internal ovule developmental events followed a sequential order. This sequential event coincided with ovule length and embryo coverage in fertilized developed ovules. Hence, based on the ovule developmental events, i.e., ovule length, embryo coverage, and endosperm nature, the classification was made to group the fruits according to size. Consequently, the fruit size can become a valuable marker for Kinnow researchers to collect the optimized fruit size for the desired ovule explant. Stages II and III had a liquid endosperm and an intact nucellus. The difference between these two stages or the shift from stage II to stage III can be confirmed with the visibility of pale green embryos to the naked eye at the micropylar end of ovules at stage III. In addition, >4-mm ovules facilitate easy handling than 2–3-mm ovules. Likewise, the completion of stage III was noticed by a change in liquid to semisolid endosperm and pink coloration at the chalazal end. After completion of stage III, fertilized and unfertilized ovules can be differentiated with uneven flatness in the ovule. Stage III fruits were found to be most suitable for explant excision (optimized size), as it could fulfill the desired ovule length, liquid endosperm, and prominent nucellus requirements.

### Somatic embryo initiation and maturation

**Figure 3:**
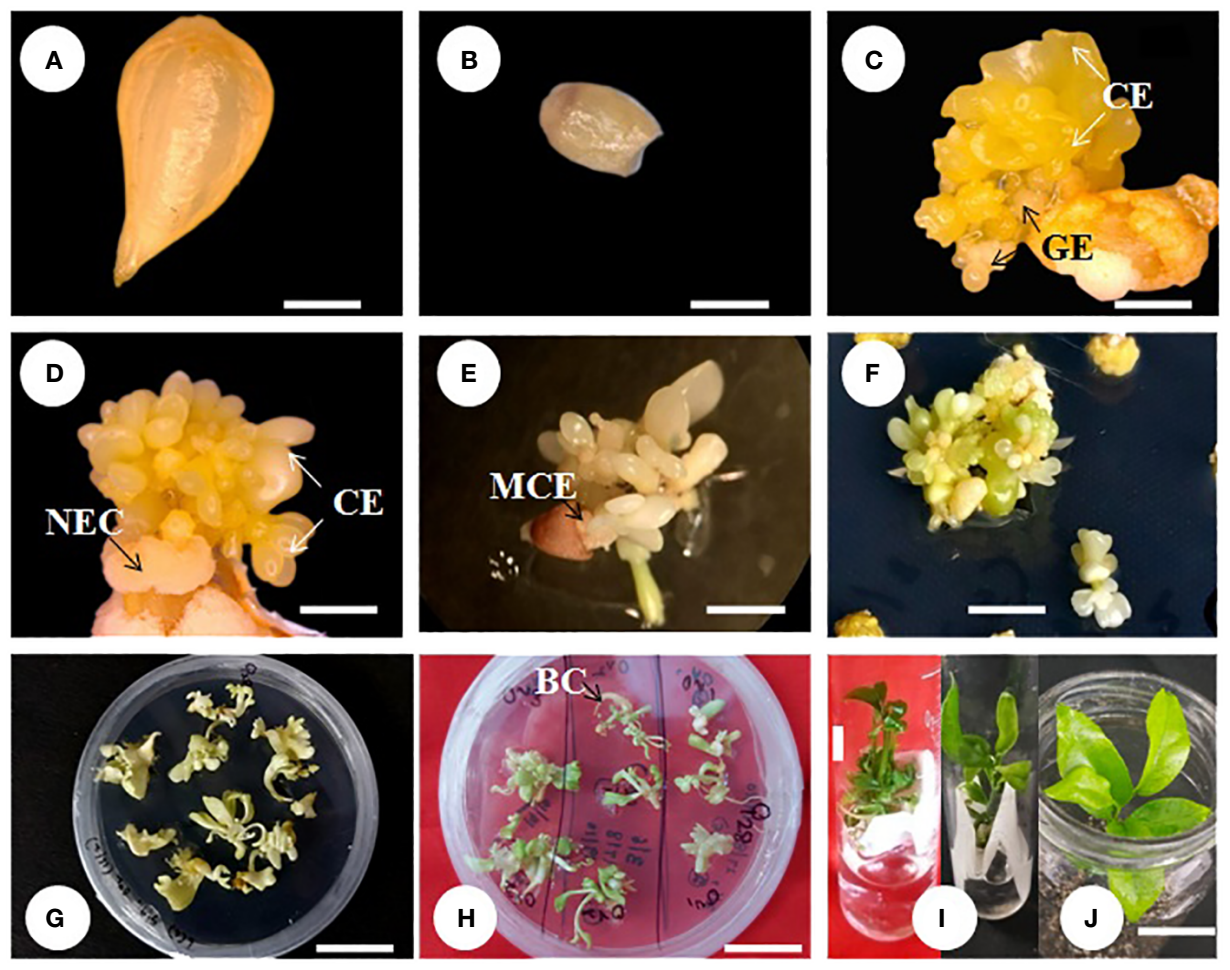
Direct somatic embryogenesis from ovule explant of Kinnow mandarin. **(A)** Stage III ovule explant, **(B)** ovule devoid of embryos, **(C–E)** somatic embryo initiation from the micropylar cut end, **(F)** proliferation of secondary embryos after subculture, **(G)** initiation of germination, **(H)** bipolar conversion, **(I)** primary plantlet establishment in liquid medium, **(J)** primary hardening. Scale bar: **(A–D)** = 1 mm, **(E)** = 3 mm, **(F–I)** = 1 cm. BC, bipolar conversion of the somatic embryo; CE, cotyledonary embryo; GE, globular embryo; MCE, micropylar cut end of explant; NEC, non-embryogenic callus.

**Figure 4:**
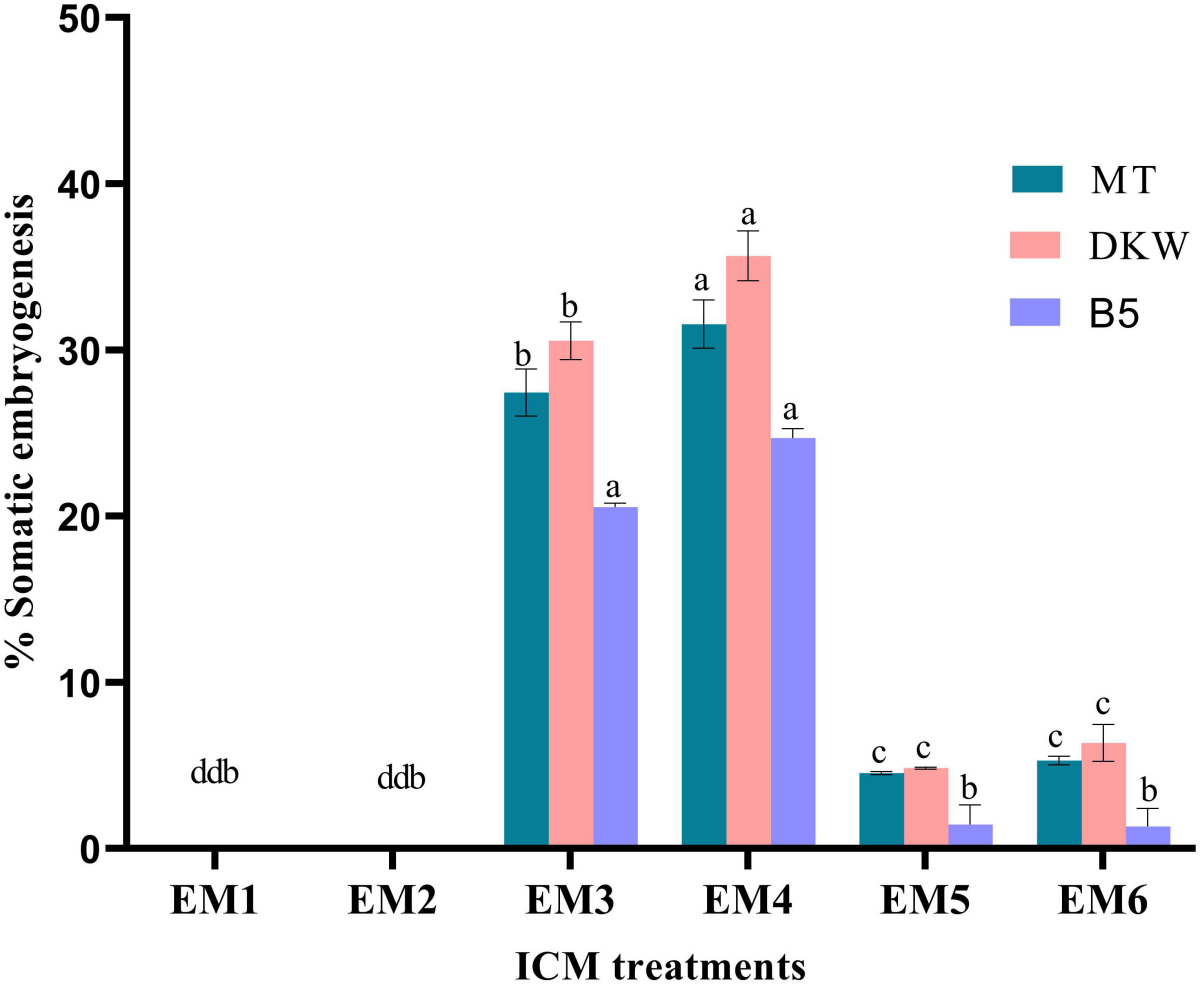
Effect of media and growth regulator combinations on somatic embryogenesis frequency in Kinnow mandarin. MT, Murashige and Tucker medium, Murashige (1969); DKW, Driver and Kuniyuki Walnut medium, Driver and Kuniyuki (1984); B_5_, Gamborg B_5_ medium Gamborg et al. (1968); EM_1_, Control; EM_2_, ME 500 mg L^-1^; EM_3_, kinetin 5.0 mg L^-1^ + ME 500 mg L^-1^; EM_4_, kinetin 5.0 mg L^-1^ + ME 1,000 mg L^-1^; EM_5_, 2,4-D 1.0 mg L^-1^ + CW 10% (v/v); EM_6_, 2,4-D 2.0 mg L^-1^ + CW 10% (v/v); ME, malt extract; CW, coconut water; ICC, induction cum maturation. Small letters denote the statistical difference; the same letters represent non-significant differences in the mean values.

Kinnow mandarin ovule explants obtained from the optimized fruit size (>21–25 mm) induced direct somatic embryos at the micropylar cut end, while pale white to yellowish non-embryogenic compact callus initiated on the outer integument of explants (**Figures 3A–E**). The ICM media containing three levels of basal media and six levels of PBR treatments were tested for DSE as given in the *Materials and Methods*. Significant differences were observed for basal media (P < 0.001), PBR treatments (P < 0.001), and their interaction (P < 0.01) (**Figure 4** **)**. Among the basal media, a higher somatic embryogenic response (12.90%) was observed on the DKW medium. Similarly, among the PBR treatments, it was significantly higher (30.64%) on EM_4_ containing cytokinin and organic additive in the form of kinetin and ME. The PBR treatments EM_1_ and EM_2_, devoid of cytokinin, failed to induce somatic embryos, but it was witnessed in the treatments EM_5_ and EM_6_ supplemented with CW. DKW and EM_4_ interaction effect revealed significantly higher (35.67%) somatic embryogenesis response compared to the other combinations on cultured *in ovulo* nucellus explants of Kinnow mandarin.

Media played an important role in inducing early somatic embryogenesis. As compared to other media tested, the earliest somatic embryogenesis (54.39 days) was recorded on the DKW medium (P < 0.001). Although PBR treatments EM_3_ and EM_4_ induced early somatic embryogenesis by maintaining statistical parity (P > 0.05) of 59.70 and 59.45 days, respectively, the overall effect of days to embryo initiation was highly significant among the PBR treatments (P < 0.001). Almost 2-fold more time was taken for somatic embryogenesis in the treatments EM_5_ and EM_6_ (**Table 1**). The interaction effect of basal media and PBR treatments, although revealed significant differences (P < 0.001), the combinations DKW × EM_3_ (56.59 days), MT × EM_4_ (56.68 days), and DKW × EM_4_ (56.21 days) recorded early somatic embryogenesis without any statistical variation.

**Table 1:** Effect of media and growth regulator combinations on somatic embryogenesis efficiency in Kinnow mandarin.

	Days to somatic embryogenesis				No. of somatic embryos per explant (at fourth month)					
Basal media ICM treatments	MT	DKW	B_5_	Marginal Mean**		MT	DKW	B_5_	Marginal Mean**	
EM_1_	0.00 ± 0.00 ^h^	0.00 ± 0.00 ^h^	0.00 ± 0.00 ^h^	0.00		0.00 ± 0.00 ^h^	0.00 ± 0.00 ^h^	0.00 ± 0.00 ^h^	0.00	
EM_2_	0.00 ± 0.00 ^h^	0.00 ± 0.00 ^h^	0.00 ± 0.00 ^h^	0.00		0.00 ± 0.00 ^h^	0.00 ± 0.00 ^h^	0.00 ± 0.00 ^h^	0.00	
EM_3_	57.44 ± 0.21^g^	56.58 ± 0.16^g^	65.06 ± 1.17^f^	59.70		51.55± 0.02^d^	87.60 ± 0.27^b^	29.26 ± 0.24^g^	56.13	
EM_4_	56.69 ± 0.22^g^	56.23 ± 0.25^g^	65.44 ± 0.11 ^f^	59.45		56.59± 0.23^c^	110.81 ± 0.67^a^	39.00 ± 0.09^f^	68.80	
EM_5_	108.33 ± 0.66^d^	106.33 ± 0.66^e^	111.33 ± 0.33^b^	108.66		35.33± 1.76^f^	37.66 ± 4.66^f^	25.66 ± 0.33^g^	32.88	
EM_6_	109.66 ± 0.33^c^	107.16 ± 0.44^de^	112.66 ± 0.66^a^	109.83		36.50± 0.50^f^	45.5 ± 2.17^e^	27.66 ± 0.33^g^	36.55	
Marginal Mean*	55.36	54.39	59.09	56.28		30.00	46.93	20.27	32.39	
LSD (P < 0.05)									
Treatment (T)	0.713				2.164				
Medium (M)	0.504				1.530				
Interaction (T × M)	1.236				3.749				

Mean data ± standard error and means with the same alphabets are not statistically different. Row mean (*) represents the media mean regardless of treatments, and column mean (**) represents the treatment mean regardless of media. LSD, least significant difference; ICM, induction cum maturation; MT, Murashige and Tucker medium Murashige (1969); DKW, Driver and Kuniyuki Walnut medium Driver and Kuniyuki (1984); B_5_, Gamborg B_5_ medium Gamborg et al. (1968); EM_1_, Control; EM_2_, ME 500 mg L^-1^; EM_3_, kinetin 5.0 mg L^-1^ + ME 500 mg L^-1^; EM_4_, kinetin 5.0 mg L^-1^ + ME 1,000 mg L^-1^; EM_5_, 2,4-D 1.0 mg L^-1^ + CW 10% (v/v); EM_6_, 2,4-D 2.0 mg L^-1^ + CW 10% (v/v); ME, malt extract; CW, coconut water; ICC, induction cum maturation.

Observations on the number of embryos per explant 4 months after embryo initiation were significantly higher (46.93) on the DKW medium (P < 0.001). Similarly, among the PBR combinations, EM_4_ yielded a significantly (P < 0.001) higher number of embryos (68.80). The interaction effect of the DKW × EM_4_ combination induced a significantly (P > 0.001) higher number of somatic embryos (110.81) per explant (**Table 1**) than other combinations (**Figures 3C–F**).

### Somatic embryo germination and bipolar conversion

**Figure 5:**
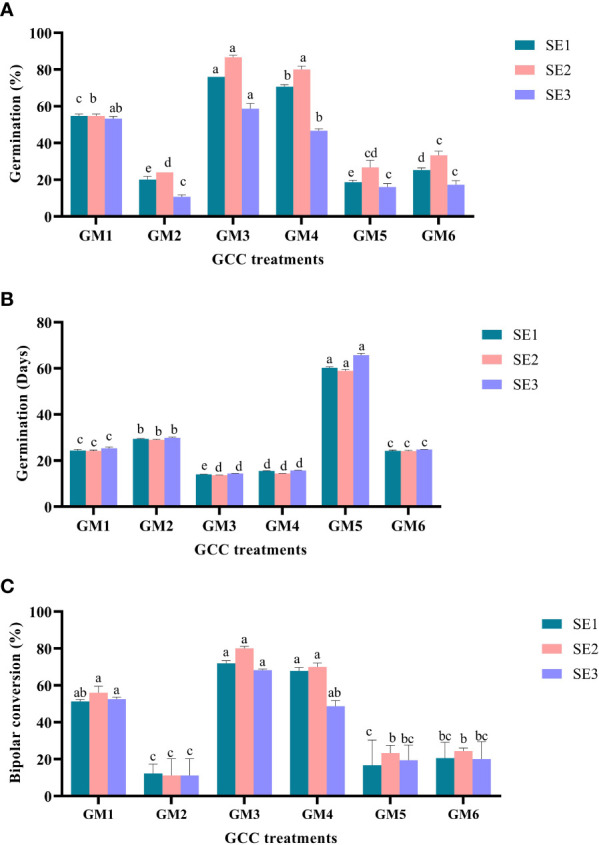
Effect of media and growth regulator composition on somatic embryo germination in Kinnow mandarin. **(A)** % germination, **(B)** days to germination, and **(C)** % bipolar conversion. Somatic embryos SE_1_ (MT × EM_4_), SE_2_ (DKW × EM_4_), and SE_3_ (B_5_ × EM_4_); GA_3_, gibberellic acid; NAA, ά-naphthaleneacetic acid; BAP, 6-benzylaminopurine; ABA, abscisic acid; AC, activated charcoal; GM_1_, Basal MT; GM_2_, MT + ME 500 mg L^-1^; GM_3_, MT + GA_3_ 2.0 mg L^-1^ + NAA 0.5 mg L^-1^ + spermidine 100 mg L^-1^ + CW 10% (v/v); GM_4_, DKW + GA_3_ 2.0 mg L^-1^ + NAA 0.5 mg L^-1^ + spermidine 100 mg L^-1^ + CW 10% (v/v); GM_5_, DKW + kinetin 5.0 mg L^-1^ + ME 1,000 mg L^-1^; GM_6_, B_5_ + BAP 2.0 mg L^-1^ + NAA 0.2 mg L^-1^ + GA_3_ 0.5 mg L^-1^ + ABA 0.2 mg L^-1^ + AC 200 + CW 10% (v/v). MT, Murashige and Tucker medium; DKW, Driver and Kuniyuki Walnut medium; B5, Gamborg B5 medium; EM1, Control; EM2, ME 500 mg L-1; EM3, kinetin 5.0 mg L-1 + ME 500 mg L-1; EM4, kinetin 5.0 mg L-1+ ME 1,000 mg L-1; EM5, 2,4-D 1.0 mg L-1 + CW 10% (v/v); EM6, 2,4-D 2.0 mg L-1 + CW 10% (v/v); ME, malt extract; CW, coconut water. Small letters denote the statistical difference; the same letters represent non-significant differences in the mean values.

Somatic embryo germination was examined by transferring three sets of somatic embryos from the ICM medium (SE_1_, SE_2_, and SE_3_) onto six treatments (GM_1_–GM_6_) of GCC medium. Significantly (P < 0.0001) higher somatic embryo germination (50.89%) was noticed in SE_2_ embryos. In contrast, to control, a similar significant trend (P < 0.001) was also obtained on GM_3_ treatment, which had a higher embryo germination of 73.78%. Furthermore, the interaction effect of SE_2_ × GM_3_ had the highest germination frequency (86.67%) and was significantly superior to the rest of the other interactions (P < 0.001) (**Figure 5A** **)**.

Early germination was observed (27.37 days) on SE_2_ embryos and GM_3_ (14.01 days) treatment (P < 0.001). The duration of embryo germination was significantly (P < 0.001) influenced by the interaction between ICM medium embryos and GCC medium treatments. However, statistical similarity (P > 0.05) was observed for early germination in the interaction effect of all three sets of embryos on GM_3_ and GM_4_ treatments. The germination duration ranged between 13.64 and 15.69 days for the above interactions (**Figure 5B** **)**.

In the present study, significantly (P < 0.001) higher bipolar conversion ensued in GM_3_ treatment (73.39%) of GCC medium and has statistical parity with GM_1_ and GM_4_ (**Figure 5C** **)**. However, ICM medium embryos and interaction between ICM medium embryos and GCC medium treatments displayed statistical parity in bipolar conversion efficiency (**Figures 3G, H** **)**.

### Primary plantlet establishment and acclimatization of emblings

**Figure 6:**
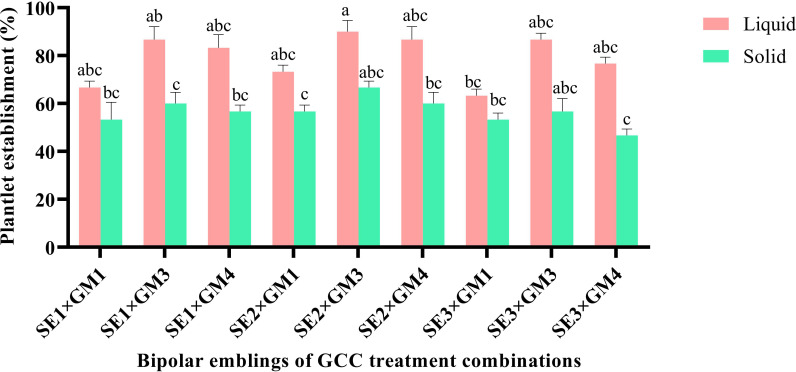
Effect of germination treatments on plantlet establishment frequency in liquid and solid MT medium in Kinnow mandarin. Somatic embryos SE1 (MT × EM4), SE2 (DKW × EM4), and SE3 (B5 × EM4); GM1, Basal MT; GM3, MT + GA3 2.0 mg L-1 + NAA 0.5 mg L-1 + spermidine 100 mg L-1 + CW 10% (v/v); GM4, DKW + GA3 2.0 mg L-1 + NAA 0.5 mg L-1 + spermidine 100 mg L-1 + CW 10% (v/v). MT, Murashige and Tucker medium; DKW, Driver and Kuniyuki Walnut medium; B5, Gamborg B5 medium; EM4, kinetin 5.0 mg L-1 + ME 1,000 mg L-1; ME, malt extract; CW, coconut water; GA3, gibberellic acid; NAA, α-naphthaleneacetic acid. Small letters denote the statistical difference; the same letters represent non-significant differences in the mean values.

Significant impact was observed in plantlet establishment when bipolar seedlings transferred from GCC media onto PBR-free establishment media. Among the bipolar converted seedlings, the highest plantlet establishment frequency was witnessed on SE_2_ × GM_3_ seedlings (78.68%); however, it was statistically on par with few other combinations tested. Among the PBR-free media, it was significantly higher (P < 0.01) in the liquid medium (79.26%) in comparison to the solid medium. The liquid medium also induced new leaf within a week with a delayed leaf emergence on the solid medium. The interaction effect of the above two, i.e., seedlings of GCC combinations and establishment medium, showed nonsignificance establishment frequency, and the emblings were ready for primary hardening within 45 days (**Figures 3I**, **6**).

Emblings from the liquid medium acclimatized well compared to those from the solid medium. The mean survival percentage was significantly higher (P < 0.0001) in the emblings raised from the liquid medium (92.22%) as compared to the solid medium. In comparison, during hardening, significantly higher survival (78.33%) was recorded on the potting medium, P_1_. The interaction effect of liquid medium × P_1_ potting medium showed cent percent plantlet survival and was significantly superior (P < 0.001) compared to other combinations (**Table 2**). Similarly, leaf emergence was early in the emblings established on liquid medium (40.80 days) and was statistically superior (P < 0.001) compared to the observations recorded on leaf emergence in emblings of solid medium. With regard to the observation on leaf emergence in potting media, it was significantly earliest (36.39 days) in P_1_ potting medium (P < 0.001) followed by P_2_. However, the interaction effect of two factors, i.e., SE_2_ × GM_3_ emblings of PBR-free establishment media and potting media, revealed nonsignificant (P > 0.05) differences for days to new leaf emergence (**Table 2**). Observations on shoot length indicated better shoot growth with significantly (P < 0.001) longer shoots on liquid medium emblings (6.38 cm) and P_1_ potting medium (6.39 cm). The interaction effect between the liquid and P_1_ potting medium combination resulted in significantly (P < 0.001) longer shoots of 7.53 cm (**Table 2**) on the 60th day. Finally, observations on root length were measured maximum (P < 0.0001) in liquid medium (9.92 cm) and P_1_ potting medium (12.15 cm). The interaction effect of these two combinations also had significantly (P < 0.001) longer roots (16.08 cm) on the 60th day (**Table 2**; **Figure 3J**).

**Table 2:** Effect of liquid and solid medium-established Kinnow mandarin plants on primary hardening in different potting media.

	% Acclimatization			Days to new leaves emergency			Shoot length (cm)			Root length (cm)		
Emblings Potting media	Liquid	Solid	Marginal Mean**	Liquid	Solid	Marginal Mean	Liquid	Solid	Marginal Mean	Liquid	Solid	Marginal Mean**
P_1_	100 ± 0.00^a^ (89.88)	56.66 ± 3.33^cd^ (44.94)	78.33	35.26 ± 0.29^e^	37.52 ± 0.07^d^	36.39	7.53 ± 0.09^a^	5.24 ± 0.12^c^	6.39	16.08 ± 0.31^a^	8.21 ± 0.28^bc^	12.15
P_2_	90 ± 0.00^c^ (48.77)	50.00 ± 0.00^b^ (68.76)	70.00	41.74 ± 0.25^c^	45.33 ± 0.63^b^	43.53	5.97 ± 0.19^b^	4.23 ± 0.07^d^	5.13	8.61 ± 0.54^b^	7.34 ± 0.27^c^	7.98
P_3_	86.66 ± 3.33^b^ (71.47)	43.33 ± 3.33^d^ (41.09)	65.00	45.38 ± 0.32^b^	49.46 ± 1.15^a^	47.42	5.61 ± 0.15^bc^	4.07 ± 0.15^d^	4.83	5.06 ± 0.53^d^	4.40 ± 0.04^d^	4.74
Marginal Mean*	92.22	50.00	71.11	40.80	44.11	42.45	6.38	4.51	5.45	9.92	6.65	8.29
LSD(P < 0.05)												
Treatment (T)	2.784			1.027			0.250			0.665		
Medium (M)	3.410			1.258			0.306			0.815		
Interaction (T × M)	4.822			1.779			0.483			1.152		

Mean data ± standard error and means with the same alphabets are not statistically different. Row mean (*) represents the media mean regardless of treatments, and column mean (**) represents the treatment mean regardless of media. Values in parentheses are arcsine transformed. LSD, least significant difference; P_1_, cocopeat:vermiculite:perlite (2:1:1); P_2_, cocopeat:vermiculite (2:1); P_3_, cocopeat:perlite (2:1).

### Histological analysis

**Figure 7:**
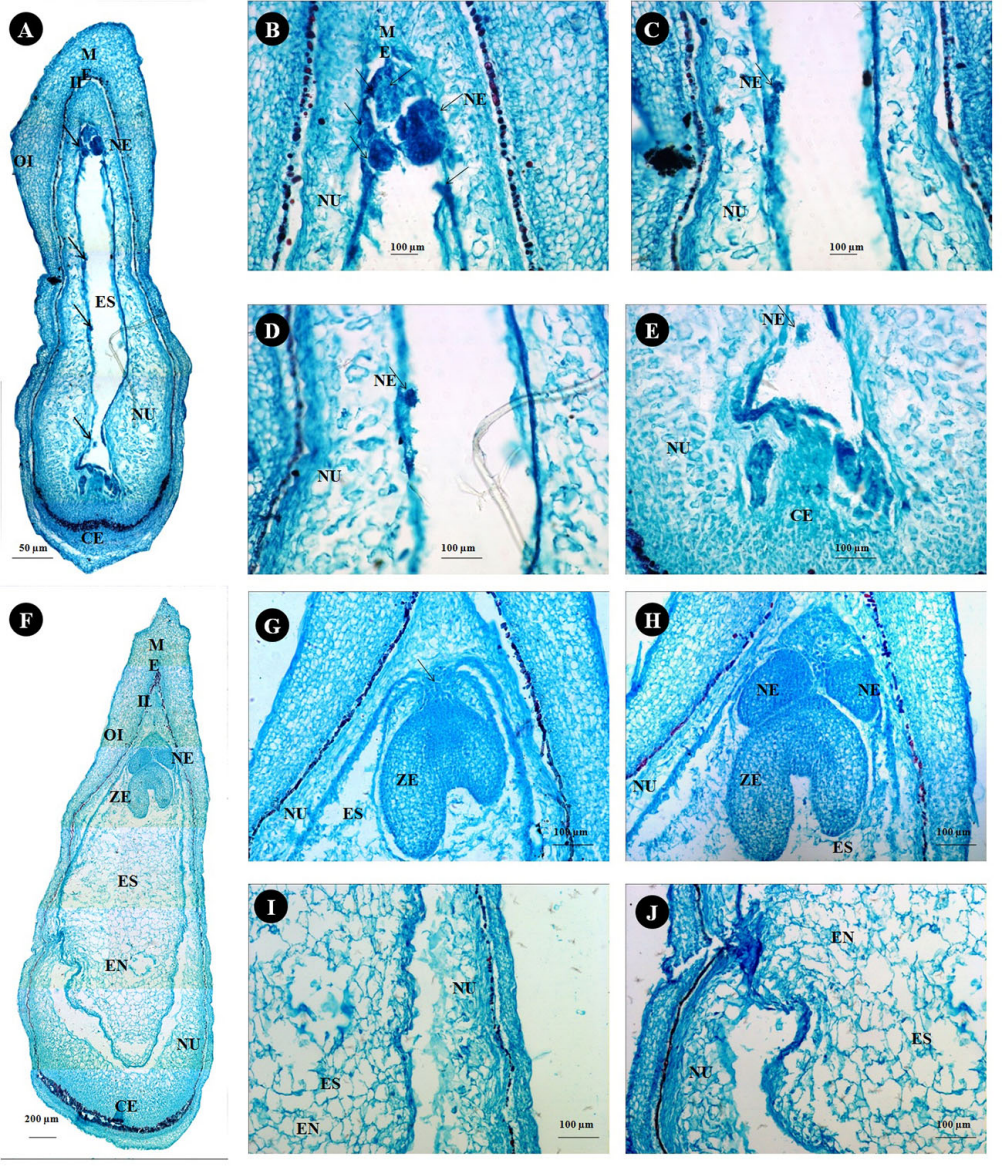
Histological study on ovule developmental events in Kinnow mandarin. **(A)** Longitudinal section of stage II ovule from 15-mm fruits (arrow indicates the nucellar embryo), **(B–E)** magnified view of a different region of panel **(A, B)**, micropylar end region; **(C, D)**, middle region; **(E)**, chalazal end region). **(F)** Longitudinal sections of stage III from 24-mm fruits, **(G)** micropylar region of stage III ovule with the zygotic embryo (arrow represents the connecting tissue), **(H–J)** magnified view of a different region of panel **(F, G)**, micropylar region; **(H)**, middle region; **(I)**, chalazal region). CE, chalazal end; EN, endosperm; ES, embryo sac; II, inner integument; ME, micropylar end; NE, nucellar embryo; NU, nucellus tissue; OI, outer integument; ZE, zygotic embryo.

**Figure 8:**
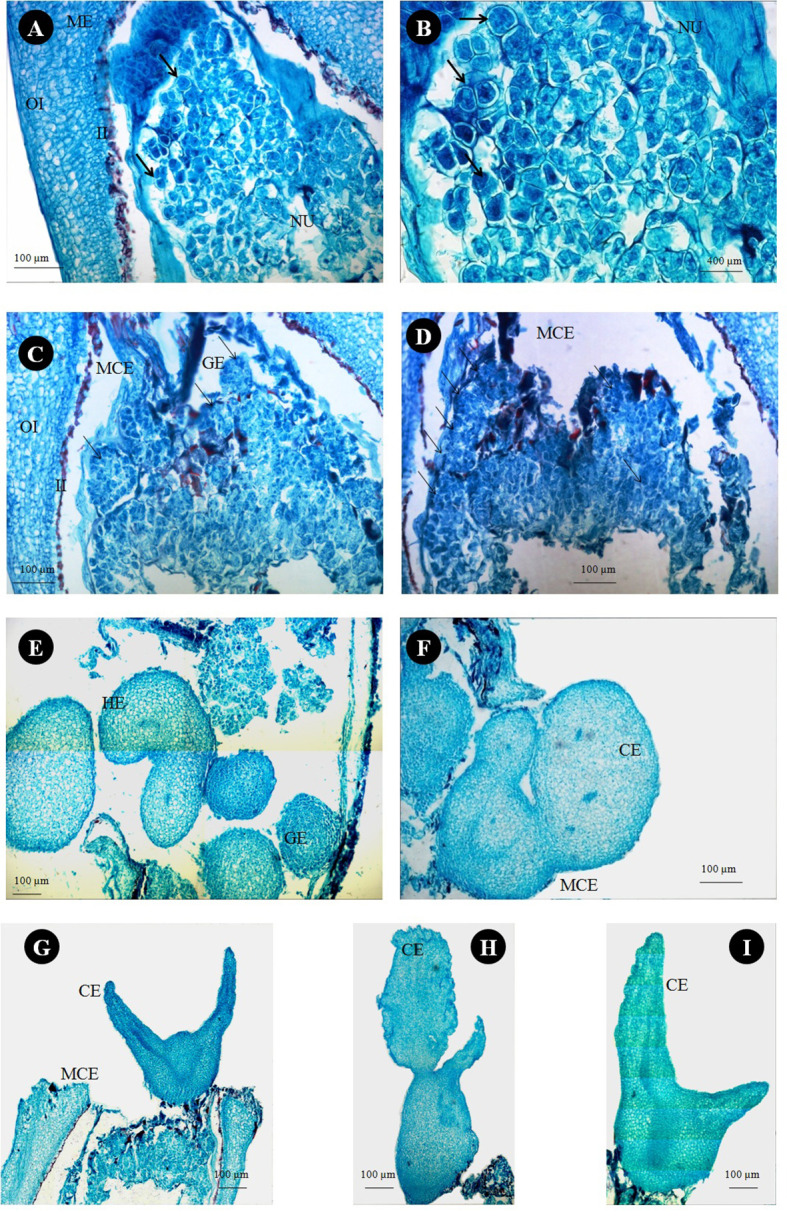
Histological study on *in vitro* somatic embryo development from stage III ovules of Kinnow mandarin inoculated after removal of preexisting embryos. **(A)** Nucellus region of stage III ovule 1.5 months after ovule inoculation on induction medium. **(B)** Magnified view of panel **(A)** (arrow represents the actively dividing embryogenic cells). **(C, D)** Globular somatic embryos at the micropylar cut end 2 months after ovule inoculation on induction medium (arrow represents globular embryos). **(E–I)** Different developmental stages of somatic embryos at the micropylar cut end of inoculated ovules. CE, cotyledonary somatic embryo; GE, globular somatic embryo; HE, heart-shaped somatic embryos; II, inner integument; MCE, micropylar cut end; ME, micropylar end; NU, nucellus tissue; OI, outer integument.

Anatomical observations of longitudinal ovule sections revealed gradual depletion of nucellus tissue with corresponding embryo sac expansion and simultaneous swift embryo growth between stages II and III (**Figures 7A, F** **)**. Observations on the existence of independent embryos within an embryo sac confirm the phenomenon of nucellar embryony in Kinnow mandarin. Globular embryos of almost similar size were observed in stage II at the micropylar end, while a few celled proglobular embryos were distributed at the micropylar half and chalazal end (**Figures 7A–E**). Dominant growth of embryo with vascular connection was observed along with almost similar size of other embryos at the micropylar end. In contrast, embryos could not be seen either at the micropylar half or the chalazal end of the nucellus or embryo sac at stage III (**Figures 7F–J**). Observations on inoculated ovules (complete removal of preexisting embryos at the time of inoculation) in the somatic embryo ICM medium (DKW × EM_4_) revealed exciting results (**Figures 8A–I**). A distinct actively dividing embryogenic cell with dense cytoplasm and a loose cellular arrangement was seen in the micropylar region at 1.5 months of culture onto ICM medium (**Figures 8A, B** **)**. The presence of subsequent developmental stages, i.e., globular, heart, and cotyledonary embryos, was observed 2 months after inoculation (**Figures 8C–I**). At the same time, compact non-embryogenic callus growth was initiated from the outer integuments.

### Genetic fidelity assessment

**Figure 9:**
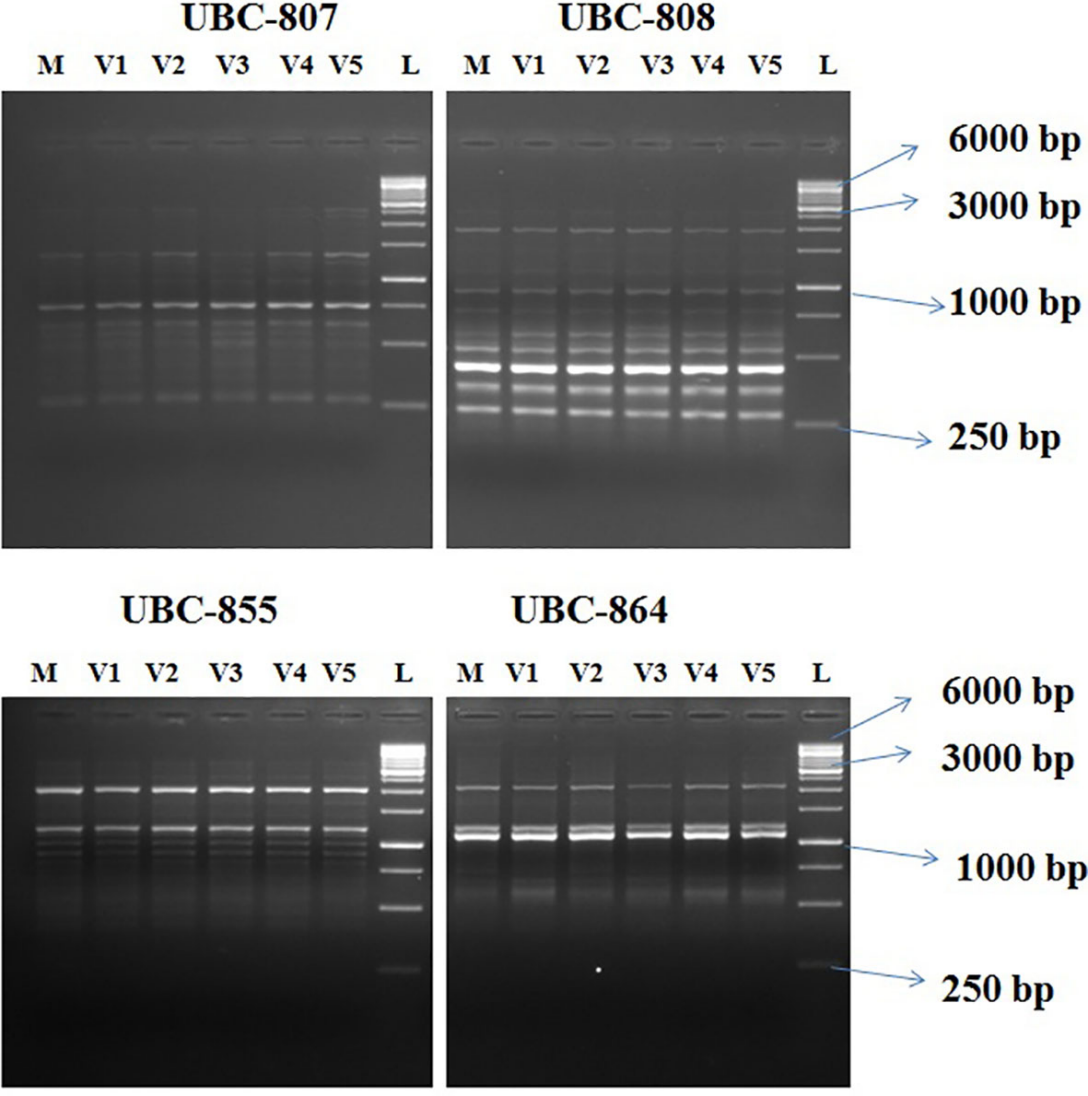
Agarose gel profile with ISSR markers UBC-807, UBC- 808, UBC- 855, and UBC-864. M, Mother plant; V1–V5, *in vitro*-regenerated plants; L, 1-Kb ladder. ISSR, Inter Simple sequence repeats; UBC, the University of British Columbia; bp, base pair.

Out of 10 ISSR markers screened, eight amplified the genomic DNA with reproducible multiple bands. The regenerated plants showed a monomorphic profile with the mother plant (**Figure 9**). Among the eight markers, UBC 855 produced the maximum of 14 reproducible monomorphic bands, followed by UBC 807 (12 bands), UBC 841 (11 bands), UBC 808 (10 bands), UBC 825 (10 bands), and UBC 827 (10 bands) (**Table 3**). The lower number of monomorphic amplification profiles was noticed from UBC 812 (five bands) and UBC 864 (six bands) ISSR markers.

**Table 3:** Details of amplification products obtained from ISSR markers in assessing clonal fidelity in Kinnow mandarin plantlets.

Sl. No.	Primer	Sequence information	No. of amplified bands	No. of polymorphic bands
1.	UBC-807	AGAGAGAGAGAGAGAGT	12	–
2.	UBC-808	AGAGAGAGAGAGAGAGG	10	–
3.	UBC-811	GAGAGAGAGAGAGAGAC	–	–
4.	UBC-812	GAGAGAGAGAGAGAGAA	5	–
5.	UBC-815	ACACACACACACACACT	10	–
6.	UBC-827	ACACACACACACACACG	10	–
7.	UBC-841	GAGAGAGAGAGAGAGAYC	11	–
8.	UBC-855	TGTGTGTGTGTGTGTGRT	14	–
9.	UBC-858	ACACACACACACACACYT	–	–
10.	UBC-864	ATGATGATGATGATGATG	6	–

ISSR, Inter Simple sequence repeats; UBC, the University of British Columbia; bp, base pair.

## Discussion

The possibility of DSE competence of the *in ovulo* nucellus culture in Kinnow mandarin was assessed in the present study to explore the regeneration potential of embryogenic cells present within the nucellus tissues. Several reports show that degeneration of the nucellus tissue coincides with the enlargement of the embryo sac; hence, the explant collection stage is important in citrus (Bitters et al., 1972; Koltunow et al., 1995; Xu et al., 2021). A weekly collection of fruits has been advocated in citrus for somatic embryogenesis (Bitters et al., 1972). However, since flowering and fruiting are region-specific and weather-dependent, identifying optimum-size fruits with a well-developed nucellus and liquid endosperm is a prerequisite for efficient *in ovulo* culture. The identification and classification of immature fruit growth stages in the present study revealed that for the stage II fruits (>14–21 mm in diameter), although provide a chance for true-to-type regeneration, complete removal of the preexisting embryos from the ovule is not feasible due to the scattered distribution of proglobular nucellar embryos. Attempts to remove the preexisting embryos at this stage may deviate from the objective of solid mutant induction because there are ample chances for chimera formation. Stage III fruits having a size between >21 and 25 mm had ovules with a well-developed intact nucellus and liquid endosperm; the proglobular embryos migrated completely to the micropylar end. It facilitated easy removal of the preexisting embryos and was convenient to handle *in vitro*. Earlier reports on nucellus culture in citrus species had also emphasized that at the time of explant collection, the ovule must have a liquid endosperm with an intact nucellus for better somatic embryogenesis (Bitters et al., 1972). There is a dearth of literature pertaining to the specific recommendation on fruit size in Kinnow mandarin for efficient ovule culture, which was accomplished in this study. Standardization of fruit size will enable researchers to directly collect the fruits at the recommended growth stage and use them for *in vitro* culture initiation.

Several factors, such as the developmental stages of the explant, culture medium, hormonal concentration, and combinations of other factors, influenced the DSE of cultured explants. In the present investigation, although preexisting embryos were eliminated entirely from the inoculated explants, somatic embryogenesis was witnessed possibly due to the stimulative effect of culture media composition. The findings correlate well with the histological observations and are in consonance with the results of the earlier workers in the nucellus culture of different citrus species (Bitters et al., 1972; Kochba et al., 1972; Navarro and Juarez, 1977) as well as mango (Wu et al., 2007). Although the use of MT medium has been widely reported in citrus somatic embryogenesis (Carimi, 2005; Dutt et al., 2010), in the present study, DKW medium stimulated a higher degree of DSE and maintained continuous proliferation of somatic embryos than the MT medium. The rapid induction and maturation of somatic embryos on DKW medium in contrast to MT medium indicate enhanced physiological activity as a consequence of reduced amounts of ammonium and chloride and increased calcium and sulfate ions as reported in citrus (Tallon et al., 2012; Tallon et al., 2013; Navarro-Garcia et al., 2016). In our study, kinetin and its combination with ME induced somatic embryos. The requirement of high concentration of cytokinin and low concentration of auxin in the medium for the induction of somatic embryos in citrus has also been suggested by Dutt et al. (2010) and Agisimanto et al. (2016). The use of 2,4-D in the culture medium of citrus species has been reported to suppress embryogenesis by inducing non-embryogenic cells due to induced osmotic stress (Pan et al., 2010; Ge et al., 2012). However, in the present study, 2,4-D in combination with CW induced somatic embryo formation in low frequency. It clearly explains the need for high cytokinin and low auxin for induction of morphogenesis, i.e., somatic embryos on nucellus tissues *via* rapid cell division and differentiation. Histological examination of the ovules in our study confirms the polyembryony nature and induction of somatic embryos through direct embryogenesis from nucellus cells in Kinnow mandarin.

*In vitro* somatic embryogenesis and polyembryony (sporophytic apomixis) can be considered artificial and natural paths of clonal multiplication mechanisms, respectively. In polyembryonic citrus species, nucellar embryony resembles somatic embryogenesis, and it is regulated by complex molecular mechanisms such as upregulation of specific genes, transposable element insertion, and gene regulation (Xu et al., 2021). Exogenous hormonal supplementation is among the other factors responsible for the expression of the above molecular signals (Mendez-Hernandez et al., 2019). Waki et al. (2011), in 2011, for the first time, demonstrated the role of the RKD gene in regulating the early developmental stages of embryogenesis in *Arabidopsis.* Subsequently, in 2018, Shimada et al. (2018) demonstrated a positive correlation between the higher expression of *citRKD1* and somatic embryogenesis in nucellus tissue of polyembryonic Satsuma mandarin. The dominant allele CitRKD1-mg2 that bears the miniature inverted-repeat transposable element (MITE)-like insertion in the upstream region is predominantly transcribed in manifesting the upregulation of *citRKD1* and is associated with polyembryony in citrus. Similarly, the repression of csSPL transcription factors (*CsSPL3* and *CsSPL14*) for initiating somatic embryogenesis in different citrus species is primarily mediated by the upregulation of miR156a (Long et al., 2018). Very recently, Feng et al. (2022) demonstrated that the binding of Fus3 and AGL15 complexes at the promoter region of miR156a is essential for its activation. Since most of the molecular regulatory pathways are conserved across the higher plants, induction of somatic embryogenesis in Kinnow possibly occurred *via* similar molecular cascade activation by hormonal stimuli. The enhanced somatic embryogenic response from *in ovulo* nucellus culture can be considered an improvement over the nucellus culture technique in mandarin, where a low embryogenic response (10%) has been reported by Bitters et al. (1972). Conversely, 22%–84% of embryo initiation has been reported from nucellus culture in sweet oranges (Kochba et al., 1972). This evidence and our experimental results suggest that mandarins have inherited *in vitro* recalcitrance. Thus, embryogenic response in citrus depends on genotype and not on the presence or absence of integuments in the developing ovules.

The practical utility of any somatic embryogenesis protocol depends on the conversion of induced embryos into healthy and quality germinated seedlings with a balanced shoot:root system (Gmitter and Moore, 1986; Corredoira et al., 2019). In our study, somatic embryos transferred from SE_2_ combination of ICM medium onto GM_3_ treatment of GCC medium gave better germination and bipolar conversion. It was possible due to the better starch and protein accumulation and post-maturation regime attained by somatic embryos from the SE_2_ combination of ICM medium. The physiological role of starch and protein accumulation for the maturation of somatic embryogenesis was demonstrated by Peng et al. (2020) in Korean pine. The molecular evidence of miR156-mediated regulation of starch accumulation during somatic embryogenesis has also been recently reported in citrus by Feng et al. (2022). Furthermore, Joshi et al. (2022) reviewed the involvement of MCM1, AGAMOUS, DEFICINS and SRF, serum response factor (MADS)-domain transcription factor *AGAMOUS-Like 15* (AGL15) in somatic embryo development by influencing functions such as transcription factors, hormone signaling, and epigenetic regulation. Our result on better germination of SE_2_ embryos correlates well with the above findings and confirms the need for the above factors for the somatic embryogenesis pathway in plant species.

Although somatic embryo maturation plays a significant role in germination, without the stimulation of meristematic activity, bipolar conversion into a complete seedling is not possible. In the present investigation, GM_3_ treatment stimulated germination and bipolar conversion in somatic embryos. A high level of three B vitamins (20.95%), i.e., thiamine, nicotinic acid, and pyridoxine, in the MT medium as compared to the DKW medium is responsible for the higher meristematic stimuli. The reports on the effect of vitamins on somatic embryo germination are scanty. However, the involvement of the same in germination as well as meristem formation has been reported by few researchers (Chen and Xiong, 2005; North et al., 2011; Ashihara et al., 2015). Moreover, the requirement of hormonal combinations for meristematic activity acquisition through enhanced cell division, cell elongation, and meristem formation in somatic embryos cannot be ignored. The positive effect of GA_3_ on shoot elongation and root initiation in citrus somatic embryos were suggested by earlier researchers (Kochba et al., 1972; Gmitter and Moore, 1986; Grosser and Gmitter, 1990; Germana, 2005). Similarly, adding CW to the germination medium on the shoot apical meristem formation and regulation of endogenous auxin levels due to substitutive cytokinin-like activity in somatic embryos was reviewed by Corredoira et al. (2019). However, the effect of cytokinin needs to be counteracted by a slight increase in auxin for balanced root:shoot growth (Corredoira et al., 2019). Hence, the enhanced bipolar conversion ratio in the present study was possible due to the addition of a lower concentration of NAA. The support of spermidine on somatic embryogenesis and meristem conversion in somatic embryos is well explained by Kaszler et al. (2021) in *Arabidopsis*. Thus, the precise combinations of the above supplements in GM_3_ medium are essential for high-frequency germination in Kinnow mandarin. Earlier studies emphasized the high vulnerability of somatic embryos toward poor germination in mandarin cultivars due to abnormal morphological defects (Benelli et al., 2010). However, in the present study, even the abnormal embryos were converted to the bipolar structure, which suggests the positive effect of GM_3_ treatment of GCC medium in Kinnow mandarin.

Efficient establishment and acclimatization of *in vitro*-raised emblings enhance survival and induce stress tolerance. In the present investigation, the germinated seedlings of SE_2_ × GM_3_ were established well in a PBR-free liquid MT medium. The phenomenal influence of GCC treatment on plantlet establishment is unclear. However, Corredoira et al. (2019), in their review on the establishment of emblings, speculated the involvement of germination medium. Preconditioning of somatic embryo germinated emblings on a hormone-free medium before transferring to a hardening medium has recently been emphasized by Asadi-Aghbolaghi et al. (2021). Our results also revealed a positive effect on growth and development of a liquid culture medium that suggests its superiority for better nutrient utilization, ease of plant removal, lesser damage to the roots, and solubilization of synthesized inhibitors near the root zone, thus resulting in the better establishment of the emblings. Spiegel-Roy and Vardi (1984) have also reported the positive effect of the liquid medium with various growth regulator combinations on the establishment of citrus species. Our protocol is an alternative wherein the hormone-free liquid medium can induce better *in vitro* establishment particularly in DSE-obtained plantlets. During primary hardening, potting medium containing cocopeat:vermiculite:perlite (2:1:1) exhibited cent percent survival of liquid medium-established plantlets due to the improved water use efficiency.

From the histological studies, it could be inferred that there is a gradual depletion of nucellus tissue during the ascending stages of fruit growth. Hence, the ideal stage of explant collection for efficient *in vitro* success is crucial (Koltunow et al., 1995; Xu et al., 2021). Our observations while studying histology revealed exciting insights. Although mandarin cultivars are reported to be facultative sporophytic apomixis polyembryony type except a few, the formation of multiple embryos through a non-apomictic pathway *via* different mechanisms such as a supernumerary bud off from the zygotic embryo, fission of a zygotic embryo, and a single ovule with two or more embryo sacs is a common phenomenon in citrus (Kepiro and Roose, 2007; Aleza et al., 2010). Hence, it is practically challenging to predict the occurrence of polyembryony based on the number of embryos. In the present study, histological observations on the existence of independent embryos other than zygotic embryo at the micropylar end, along with the migration of a few embryos at the micropylar half, clearly suggest the occurrence of sporophytic apomixis with nucellar adventive embryony in Kinnow mandarin. Suppression of zygotic embryo by nucellar embryo is the usual hypothesis in citrus, but in the present study, embryo connected with endosperm (zygotic embryo) was larger than the other embryos. Since the connection between the embryo and the endosperm is the unique distinguishable character for identifying zygotic embryos (Wakana and Uemoto, 1987), it can be concluded that Kinnow mandarin has zygotic dominance capability and hence can be used as a female parent in the breeding program.

Avoidance of nucellar and zygotic embryos is a prerequisite for obtaining solid mutants *via* single-cell and true-to-the-type regeneration. The histological observation could decipher stage III (>21–25 mm) of fruit growth as the best explant (ovule) collection stage for easy removal of preexisting embryos. The reason is the complete migration of proglobular embryos at the micropylar end. Similar were the observations of Wakana and Uemoto (1987) in a few mandarin cultivars. From the nucellus tissue of inoculated explants (stage III), induced embryogenic cells were witnessed at the micropylar end. The induced embryogenic activity was confirmed by a dense cytoplasm and loose arrangement of cells from surrounding cells with active cell division. The division of cells and formation of embryos further confirmed the single-cell regeneration and fulfilled the requirement for inducing solid mutants through the standardized protocol. In addition to the above, the normal developmental events, such as globular to cotyledonary somatic embryos, showed the efficiency of the induction medium on Kinnow somatic embryogenesis. The results confirm the hypothesis of Kobayashi et al. (1979), who advocated that any nucellus cell of embryogenic capacity can give rise to embryos.

Assessment of genetic stability of *in vitro*-grown plants is an unavoidable requirement due to the variability enforced on the regenerated plants by culture environments such as epigenetic changes, transposon activation, or chromosomal aberrations. Emphasis on using various molecular markers for genetic fidelity assessment has gained importance during the last few decades due to innumerable advantages of early and nondestructive detection of variation. The ISSR markers did not reveal any variation in the established emblings, thus confirming genetic stability with the current protocol. In citrus, earlier studies also suggest the efficiency of ISSR markers in assessing genetic stability (Siragusa et al., 2007; Meziane et al., 2017; Devi et al., 2021). The current study is the first report on the genetic stability of *in ovulo* culture-induced plants obtained through DSE in Kinnow mandarin.

## Conclusion

The outcome of the detailed investigation suggests that *in ovulo* nucellus culture can be an alternative to the nucellus culture technique in Kinnow mandarin. The size-specific recommendation, i.e., ovules of Kinnow mandarin obtained from stage III (>21–25 mm in diameter) fruits can induce DSE when inoculated on somatic embryo ICM medium containing DKW supplemented with kinetin 5.0 mg L^-1^ and ME 1,000 mg L^-1^. Transfer of cotyledonary embryos from the above medium to GCC medium containing MT basal medium supplemented with GA_3_ 2.0 mg L^-1^ + NAA 0.5 mg L^-1^ + spermidine 100 mg L^-1^ + CW 10% resulted in higher germination. Preconditioning germinated seedlings in the liquid medium revealed maximum plant establishment and cent percent survival on a potting medium containing cocopeat:vermiculite:perlite (2:1:1). The occurrence of embryogenesis from single nucellus cells confirms its practical utility in *in vitro* mutation studies for induction of solid mutants. Since the ISSR markers reveal the genetic fidelity of regenerated plants, the developed protocol can be effectively utilized not only for mutagenesis study but also for mass multiplication, virus elimination, synthetic seed technology, *in vitro* conservation, gene editing, and germplasm exchange.

## Data availability statement

The raw data supporting the conclusions of this article will be made available by the authors, without undue reservation.

## Author contributions

OA conceived the idea, TM and OA designed the study, collected data and wrote the manuscript. TM, GJ, and SK performed data analysis; SS reviewed the manuscript. GC and TM conducted histological studies. AS and TM assessed the molecular marker based genetic fidelity. All the authors contributed equally to the revision and accepted the conclusion. All authors contributed to the article and approved the submitted version.
